# Medroxyprogesterone acetate vs. GnRH antagonist for preventing premature LH surge during ovarian stimulation in assisted reproductive technology: a retrospective cohort study

**DOI:** 10.3389/frph.2026.1793207

**Published:** 2026-03-19

**Authors:** María M. Jimenez, Cristian Jesam, Karina Sequeira, Karina Alcantar

**Affiliations:** 1 Hospital La Florida Dra. Eloísa Díaz, Santiago, Chile; 2 SGFertility, Santiago, Chile

**Keywords:** assisted reproductive technology, GnRH antagonist, medroxyprogesterone acetate, PPOS protocol, progestin-primed ovarian stimulation

## Abstract

**Objective:**

To compare the effectiveness of medroxyprogesterone acetate (MPA) vs. cetrorelix (GnRH antagonist) in preventing premature LH surge during ovarian stimulation in assisted reproductive technology (ART) cycles, including cycles for intracytoplasmic sperm injection (ICSI) and fertility preservation.

**Design:**

Single-center, retrospective observational cohort study.

**Setting:**

Private fertility clinic in Santiago, Chile.

**Patients:**

A total of 2,964 ART cycles conducted between January 2018 and December 2022 were included after excluding 425 cycles that were cancelled. Among these, 1,793 were ICSI cycles (1,529 antagonist, 264 MPA) and 1,171 were fertility preservation cycles (862 antagonist, 309 MPA).

**Interventions:**

Ovarian stimulation using recombinant FSH and/or menotropins with ovulatory suppression by either oral MPA (10 mg/day) or daily cetrorelix (0.25 mg).

**Main outcome:**

Follicular response, oocyte yield, embryo development, euploidy and mosaicism rates, implantation, clinical pregnancy, miscarriage, and live birth outcomes.

**Results:**

In fertility preservation cycles, MPA produced higher numbers of follicles ≥17 mm (8.7 vs. 6.7), oocytes retrieved (12.2 vs. 10.7), and mature oocytes (9.1 vs. 7.9; *p* < 0.01 for all). In ICSI PGT-A cycles, MPA was associated with a higher number of biopsied blastocysts and frozen embryos per patient, while euploid, implantation, and live birth rates were comparable to the antagonist group. Cancellation, fertilization, and miscarriage rates did not differ significantly.

**Conclusions:**

MPA is a safe and effective oral alternative to GnRH antagonists for LH suppression during ovarian stimulation in ART, providing comparable reproductive outcomes while reducing treatment burden.

## 1 Introduction

Controlled ovarian stimulation (COS) is a cornerstone of assisted reproductive technology (ART), aiming to recruit multiple follicles while preventing premature luteinizing hormone (LH) surges. Gonadotropin-releasing hormone antagonist (GnRH-ant) protocols have become standard practice due to their convenience, predictable pituitary suppression, and low risk of ovarian hyperstimulation syndrome (OHSS) (1). However, the requirement for daily injections and their relatively high cost may negatively affect patient compliance and treatment accessibility (2).

Progestin-primed ovarian stimulation (PPOS) has emerged as an oral, injection-free alternative compatible with freeze-all strategies (3–5). In this approach, pituitary LH suppression is achieved through daily administration of an oral progestin during stimulation, with embryo transfer deferred to a subsequent hormonally controlled cycle. Since its initial description, PPOS has been shown to effectively prevent premature LH surges while maintaining satisfactory follicular recruitment and oocyte competence (3, 6).

Among available progestins, medroxyprogesterone acetate (MPA) has been extensively investigated and demonstrated reproductive outcomes comparable to those of GnRH-antagonist protocols, even at lower doses (7–9). Randomized controlled trials and large observational studies evaluating both dydrogesterone and MPA have consistently demonstrated non-inferiority with respect to ovarian response, embryo development, and live birth outcomes, suggesting a potential class effect of oral progestins (7–10).

Comparative studies across diverse clinical indications—including oocyte donation, autologous IVF/ICSI cycles, polycystic ovary syndrome (PCOS), and fertility preservation—generally report equivalent or, in some cases, superior outcomes with PPOS (7–12). In women with PCOS or high ovarian response, PPOS has also been associated with effective LH suppression and a low risk of OHSS (12).

In fertility preservation, PPOS offers distinct logistical and patient-centered advantages. Because embryo transfer is not planned in these cycles, oral LH suppression eliminates the need for injectable medications and allows flexible or random-start stimulation, which is particularly advantageous for oncologic patients or those requiring urgent treatment initiation (4, 5, 13). Similarly, in ICSI cycles incorporating preimplantation genetic testing for aneuploidy (PGT-A), several studies have reported comparable blastulation rates, euploidy rates, and live birth outcomes between PPOS and GnRH-antagonist protocols when a freeze-all strategy is applied (10, 11).

Importantly, neonatal outcomes following PPOS appear reassuring, with no increased risk of prematurity, low birth weight, or congenital anomalies reported to date (14, 15). Nevertheless, uncertainties remain regarding the consistency of MPA-based protocols across different clinical indications and whether specific differences—such as gonadotropin requirements, blastocyst yield, or rates of embryonic mosaicism—may influence clinical outcomes (11, 16). Therefore, the present study aimed to compare MPA-based PPOS and GnRH-antagonist protocols in fertility preservation and ICSI cycles with PGT-A under a uniform freeze-all policy.

## 2 Materials and methods

**Figure 1:**
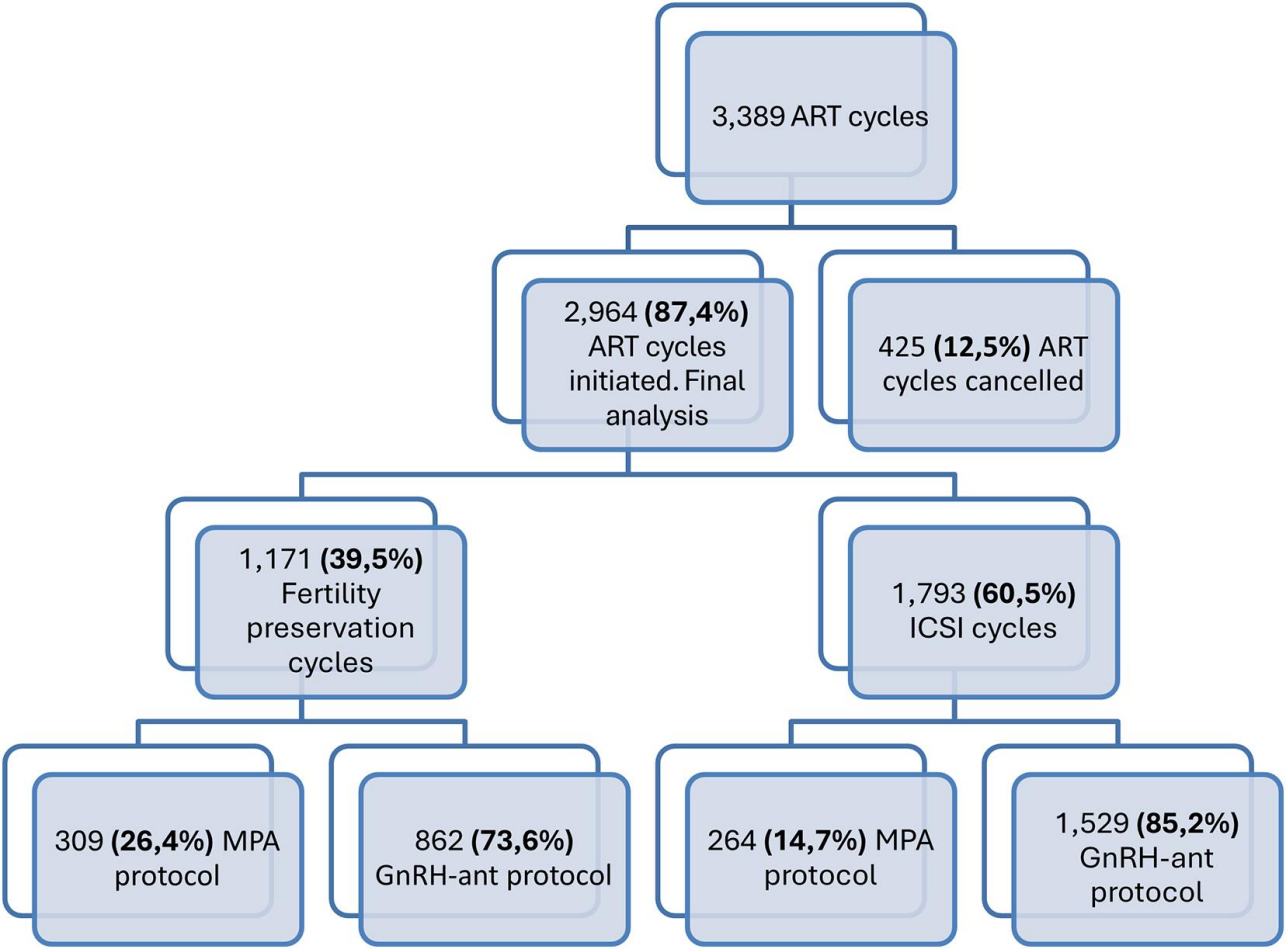
Study design.

The study design is shown in Figure 1. This single-center, retrospective observational cohort study was conducted at a private fertility clinic in Santiago, Chile, between January 2018 and December 2022. During this period, 3,389 assisted reproductive technology (ART) cycles were initiated. Of these, 425 cycles (12.5%) were canceled, and 2,964 cycles (87.4%) were included in the final analysis. Among the analyzed cycles, 1,793 corresponded to intracytoplasmic sperm injection (ICSI), of which 1,529 (85.2%) used a gonadotropin-releasing hormone antagonist (GnRH-ant) protocol and 264 (14.7%) used medroxyprogesterone acetate (MPA). In addition, 1,171 fertility preservation cycles were performed, including 862 (73.6%) GnRH-ant and 309 (26.4%) MPA cycles.

All patients provided written informed consent for treatment and for the use of de-identified clinical data for research purposes. The study protocol was approved by the institutional review board of SGFertility and conducted in accordance with the Declaration of Helsinki.

Patients undergoing ovarian stimulation for fertility preservation included women electing to delay childbearing for social reasons, individuals scheduled to receive gonadotoxic therapy, and women seeking oocyte accumulation due to diminished ovarian reserve (DOR). Patients undergoing ICSI cycles presented with a variety of infertility diagnoses. Preimplantation genetic testing for aneuploidy (PGT-A) was offered in selected cases, including advanced maternal age (>38 years), recurrent implantation failure, recurrent miscarriage, unexplained infertility, severe male factor infertility, or a family history of genetic disorders.

Baseline characteristics included age, body mass index (BMI), antral follicle count (AFC), and serum anti-Müllerian hormone (AMH) levels. Stimulation characteristics—total gonadotropin dose, duration of stimulation, and menotropin dose—were also analyzed. Missing data was not imputed, and all analyses were performed on complete available cases.

At baseline, transvaginal ultrasound was performed to confirm ovarian quiescence. Controlled ovarian stimulation was initiated between cycle days 2 and 4 using recombinant follicle-stimulating hormone (rFSH) (Gonal-F®, Pergoveris®, Rekovelle®) and/or human menopausal gonadotropins (Menopur®), with individualized dose adjustments based on follicular response. Serial transvaginal ultrasounds were performed throughout the stimulation period, which typically lasted 10–14 days.

MPA-PPOS protocol: Patients received medroxyprogesterone acetate (MPA) 10 mg orally once daily, starting on the first day of stimulation and continuing until the day of ovulation trigger.

GnRH-antagonist protocol: Patients received cetrorelix acetate (Cetrotide®) 0.25 mg subcutaneously once daily, initiated when the leading follicle reached approximately 13 mm in diameter and continued until the trigger day.

Final oocyte maturation was triggered when most follicles reached a diameter of ≥18 mm. Trigger agents included recombinant human chorionic gonadotropin (r-hCG; Ovidrel® 250 μg) or, in patients at high risk for ovarian hyperstimulation syndrome (OHSS), a gonadotropin-releasing hormone agonist (Gonapeptyl® 0.2 mg). Oocyte retrieval was performed 36 h after trigger under transvaginal ultrasound guidance and intravenous sedation. Retrieved oocytes were denuded 1.5 h later and assessed for nuclear maturity by experienced embryologists.

In fertility preservation cycles, mature (metaphase II) oocytes were vitrified 40 h post-trigger using the Cryotec® method and stored in liquid nitrogen at −196 °C under continuous laboratory monitoring.

For ICSI cycles, sperm samples were obtained from partners or donors (fresh or frozen) via ejaculation or microsurgical aspiration, as clinically indicated. ICSI was performed 2–3 h after denudation. Fertilization was assessed 16–18 h later, and embryos were cultured to the blastocyst stage (day 5–6). Blastocyst quality was evaluated using modified Gardner criteria, based on blastocoel expansion and the morphology of the inner cell mass and trophectoderm. Blastocysts were either vitrified, transferred fresh, or biopsied for PGT-A.

In PGT-A cycles, trophectoderm biopsy was performed on day 5–6 blastocysts. Between four and six cells were collected and analyzed using next-generation sequencing (NGS) on the Igenomix platform. Following biopsy, blastocysts were vitrified pending genetic results. Euploid embryos were subsequently thawed and transferred into hormonally prepared cycles.

Frozen embryo transfers (FETs) were conducted in artificial cycles. Endometrial preparation consisted of oral estradiol administration until a thickness of 7–8 mm was achieved, followed by intravaginal micronized progesterone and oral dydrogesterone for luteal support. Embryo transfer was performed after six full days of progesterone exposure. Biochemical pregnancy was defined by a positive serum β-hCG test 10 days post-transfer, and clinical pregnancy was confirmed by ultrasound visualization of a gestational sac at 6 weeks of gestation. Live births were verified through direct patient follow-up.

Primary outcomes included: (1) number of follicles ≥17 mm; (2) number of oocytes retrieved and mature (MII) oocytes; (3) embryo development and blastocyst formation; (4) euploidy, mosaicism, and aneuploidy rates; and (5) clinical outcomes, including implantation, clinical pregnancy, miscarriage, and live birth per transfer. Secondary outcomes included stimulation duration, gonadotropin consumption, and cycle cancellation rates. In fertility preservation subgroup analyses were performed according to age (≤35 vs. >35 years).

Statistical analyses were performed using GraphPad Prism version 10 (GraphPad Software, USA). Continuous variables were summarized as mean ± standard deviation (SD) and 95% confidence intervals (CI). Normality was assessed using the D'Agostino–Pearson, Anderson–Darling, Shapiro–Wilk, and Kolmogorov–Smirnov tests. Between-group comparisons for non-normally distributed variables were conducted using the Mann–Whitney U test. Categorical variables were analyzed using Fisher's exact test. A two-sided *p*-value <0.05 was considered statistically significant.

## 3 Results

### 3.1 Fertility preservation cycles

Baseline characteristics are presented in Table 1. No statistically significant differences were observed between the MPA and GnRH-antagonist groups with respect to age, body mass index (BMI), antral follicle count (AFC), anti-Müllerian hormone (AMH) levels, or duration of stimulation. Although total gonadotropin consumption was comparable overall, the GnRH-antagonist group required a slightly lower total gonadotropin dose compared with the MPA group (*p* = 0.0002).

**Table 1:** Comparative outcomes in ovarian stimulation for fertility preservation according to age in years.

Outcomes	GnRHa (*n* = 862)	MPA (*n* = 309)	*P*-value
Age, years	34.9 (± 4.5)	34.5 (± 4.5)	0.059
BMI (kg/m2)	23.6 (± 3.7) *(n* *=* *808)*	23.9 (± 3.6) *(n* *=* *296)*	0.325
AFC (*n*)	10.9 (± 6.1) *(n* *=* *789)*	10.9 (± 5.8) *(n* *=* *303)*	0.723
AMH (ng/mL)	1.91 (± 2.5) *(n* *=* *465)*	1.93 (± 1.9) *(n* *=* *226)*	0.272
Total gonadotrophin dose (IU)	2,121 (± 944.6)	2,263 (± 877.8)	0.0002***
Total menotropins dose (IU)	1,439 (± 800.5)	1,412 (± 771.6)	0.883
Days of stimulation (*n*)	10.3 (± 2.04) *(n* *=* *845)*	10.2 (± 1.8) *(n* *=* *309)*	0.248
Follicles >17 mm, *n*	6.7 (± 5.3)	8.7 (± 6.2)	<0.0001****
Oocytes retrieved, *n*	10.7 (± 8.4)	12.2 (± 8.7)	0.002**
Oocytes MII, *n*	7.9 (± 6.5)	9.1 (± 7.1)	0.002**
Cancelled cycles, *n* (%)^a^	88 (9.2%)	33 (9.6%)	0.829
Lack/inadequate ovarian response	80 (90.9%)	25 (75.7%)	0.037*
Early ovulation	2 (2.2%)	1 (3.0%)	>0.999
Medical reason/patient decision	5 (5.6%)	3 (9.1%)	0.682
Altered exams	1 (1.1%)	4 (12%)	0.019*
	≤ 35 years		
Outcomes	GnRHa (*n* = 414)	MPA (*n* = 163)	*P*-value
Age, years	31.7 (± 4.3)	31.5 (± 4.3)	0.39
Follicles >17 mm, *n*	7.7 (± 6.0)	9.4 (± 6.4)	0.0005***
Oocytes retrieved, *n*	12.6 (± 9.3)	14.0 (± 9.3)	0.064
Oocytes MII, *n*	9.4 (± 7.3)	10.5 (± 7.6)	0.566
	> 35 years		
Outcomes	GnRHa (*n* = 448)	MPA (*n* = 146)	*P*-value
Age, years	37.9 (± 1.7)	37.7 (± 1.7)	0.31
Follicles >17 mm, *n*	5.8 (± 4.3)	7.9 (± 5.9)	<0.0001****
Oocytes retrieved, *n*	9.1 (± 7.1)	10.4 (± 7.5)	0.02*
Oocytes MII, *n*	6.6 (± 5.5)	7.7 (± 6.2)	0.035*

Values are mean ± SD. *P*-values are shown: *p < 0.05, **p < 0.01, ***p < 0.001, *****p < 0.0001.

GnRHa, GnRH antagonist; MPA, medroxyprogesterone acetate; MII, metaphase II.

a Cancellation data were not included in the outcomes analysis.

Patients stimulated with MPA demonstrated significantly higher follicular and oocyte yields. The mean number of follicles ≥17 mm was significantly greater in MPA cycles than in GnRH-antagonist cycles (*p* < 0.0001). Similarly, the total number of oocytes retrieved and the number of mature (metaphase II, MII) oocytes were significantly higher in the MPA group.

Age-stratified analyses revealed consistent patterns across age categories. Among younger patients (≤35 years), MPA cycles were associated with a higher number of follicles ≥17 mm, whereas in older patients (>35 years), MPA cycles yielded higher total and mature oocyte numbers. Overall cycle cancellation rates were comparable between protocols; however, cancellations due to inadequate ovarian response occurred more frequently in the GnRH-antagonist group (*p* = 0.037).

### 3.2 ICSI-PGT-A cycles

Baseline demographic and ovarian reserve characteristics were similar between the MPA and GnRH-antagonist groups (Table 2). The MPA group exhibited a higher number of follicles ≥17 mm and a greater total number of oocytes retrieved per cycle. In contrast, the number of mature (MII) oocytes and fertilization rates did not differ significantly between protocols.

**Table 2:** Comparative outcomes in ovarian stimulation and ICSI data in preimplantation genetic testing for aneuploidy.

Outcomes	GnRHa (*n* = 1,529)	MPA (*n* = 264)	*P*-value
Age, years	36.4 (± 3.8)	36.9 (± 3.7)	0.073
BMI (kg/m2)	24.4 (± 3.7) *(n* *=* *1,468)*	24.5 (± 3.3) (*n* = 248)	0.305
AFC (n)	11.5 (± 6.5) *(n* *=* *1,360)*	11.9 (±6.6) *(n* *=* *230)*	0.356
AMH (ng/mL)	2.12 (± 2.4) *(n* *=* *905)*	2.12 (± 2.1) *(n* *=* *190)*	0.775
Total gonadotrophin dose (IU)	2,161 (± 796)	2,201 (± 1,039)	0.548
Total menotropins dose (IU)	1,248 (± 573)	1,307 (± 521)	0.009
Days of stimulation (*n*)	11.2 (± 1.9) *(n* *=* *1,497)*	11.2 (± 1.7) *(n* *=* *263)*	0.937
Follicles >17 mm, *n*	7.4 (± 5.1)	9.1 (± 5.6)	<0.0001****
Oocytes retrieved, *n*	11.8 (± 8.3)	13.1 (± 9.2)	0.032*
Oocytes MII, *n*	9.3 (± 6.8)	10.1 (± 7.4)	0.1002
Fertilized oocytes, *n*	6.9 (± 4.8)	7.4 (± 5.0)	0.131
Two pronuclei, *n*	6.4 (± 4.5)	6.9 (± 4.8)	0.139
Embryo, *n*	6.4 (± 4.5)	6.9 (± 4.8)	0.151
Embryo A, *n*	0.3 (± 0.7)	0.2 (± 0.5)	0.048*
Embryo B, *n*	1.1 (± 1.3)	1.2 (± 1.4)	0.006**
Embryo C, *n*	0.8 (± 1.1)	1.2 (± 1.3)	<0.0001****
Embryo D, n	0.1 (± 0.5)	0.3 (± 0.6)	0.001***
Blastocyst biopsied per patient, n	2.7 (± 2.1)	3.5 (± 2.3)	<0.0001****
Frozen embryo per patient, *n*	2.1 (± 2.3)	2.7 (± 2.4)	0.0001***
Aneuploid rate per biopsied blastocyst, %	45.9	44.3	0.507
Euploid rate per biopsied blastocyst, %	47.1	46.3	0.741
Mosaic no transferable rate per biopsied blastocyst, %	0.8	2.2	0.007**
Mosaic transferable rate per biopsied blastocyst, %	2.3	5.5	0.0002***
Disease-carry rate per biopsied blastocyst, %	0.18	0	0.56
Amplification failure per biopsied blastocyst, %	0.18	0.4	0.353
No data (re-biopsy) per biopsied blastocyst, %	2.9	1.1	0.013*
Thawed Embryo Survival rate, %	91.8 (*n* = 1,307)	95.1 (*n* = 194)	0.109
Clinical pregnancy rate, %	56.8	63.1	0.120
Implantation rate, %	53.4	60.1	0.085
Miscarriage rate, %	16.02	12.6	0.398
Live birth rate (per embryo transfer), %	45.6	52.3	0.105
Live birth rate (per embryo transferred), %	41.5	48.9	0.057
Cancelled cycles, *n* (%)^a^	257 (14.4%)	47 (15.1%)	0.727
Lack/inadequate ovarian response	212 (82.5%)	39 (82.9%)	>0.999
Early ovulation	8 (3.1%)	0 (0%)	0.614
Medical reason/patient decision	16 (6.2%)	5 (10.6%)	0.342
Altered exams	21 (8.2%)	3 (6.4%)	>0.999

Mann–Whitney test was used for quantitative variables, Fischer's exact test was used to compare proportions.

GnRHa, GnRH antagonist. MPA, medroxyprogesterone acetate.

Values are presented as mean ± SD or %. *p* values are shown: *p < 0.05, ** *p* < 0.01, ***p < 0.001, *****p < 0.0001.

a Cancellation data were not included in the outcomes analysis.

Embryologic outcomes favored MPA cycles in terms of the number of blastocysts available for biopsy and cryopreservation per patient. Mosaic blastocysts were observed more frequently in the MPA group, whereas euploid and aneuploid rates were comparable between protocols. Importantly, clinical outcomes, including implantation rate, clinical pregnancy rate, and live birth rate per transfer, were statistically equivalent between MPA and GnRH-antagonist protocols.

## 4 Discussion

This retrospective cohort study compared progestin-primed ovarian stimulation using medroxyprogesterone acetate (MPA-PPOS) with conventional gonadotropin-releasing hormone antagonist (GnRH-ant) protocols in two distinct clinical populations: fertility preservation (FP) and intracytoplasmic sperm injection (ICSI) cycles incorporating preimplantation genetic testing for aneuploidy (PGT-A). Under a uniform freeze-all strategy, MPA-PPOS achieved reproductive outcomes comparable to those of GnRH-ant protocols in terms of LH suppression, follicular recruitment, embryo development, and clinical outcomes, in agreement with previous randomized trials and large observational studies (7–11).

In our study, the GnRH- ant protocol was administered in a flexible manner once the leading follicle reached >13 mm in diameter. Serum LH levels were not routinely assessed on the day of antagonist initiation, which limits our ability to evaluate the degree of LH suppression in individual patients. Excessive LH suppression in women with low baseline LH concentrations (<4 IU/L) may negatively affect reproductive outcomes, as previously suggested by Liu et al. (12). Although this hypothesis cannot be confirmed in our cohort, it represents a potential biological explanation for outcome variability observed in antagonist cycles.

In fertility preservation cycles, MPA-PPOS was associated with a higher number of follicles ≥17 mm, as well as increased total and mature (MII) oocyte yield. These findings were generally maintained across age groups. However, when stratified by age, the observed difference was confined to the number of follicles ≥17 mm, without significant differences in total oocytes retrieved or MII oocytes. This suggests that the apparent advantage of MPA-PPOS may be primarily related to follicular growth dynamics rather than a consistent improvement in oocyte yield. As expected, younger patients (<35 years) demonstrated higher oocyte recovery compared with those ≥35 years, irrespective of stimulation protocol, reflecting the well-established impact of age on ovarian response.

Stimulation duration and overall cancellation rates were similar between protocols, although MPA cycles required a modestly higher gonadotropin dose, a finding previously described in PPOS protocols (3, 7). Importantly, baseline ovarian reserve parameters were comparable between groups, suggesting that the observed differences in oocyte yield are unlikely to be explained by baseline reserve imbalance.

In ICSI–PGT-A cycles, MPA-PPOS was associated with a higher number of blastocysts available for biopsy and cryopreservation, while euploidy and aneuploidy rates were similar between protocols. Implantation, clinical pregnancy, and live birth rates were equivalent, corroborating previous randomized and meta-analyses demonstrating non-inferiority of PPOS strategies under freeze-all conditions (10, 11).

A slightly higher proportion of mosaic blastocysts was observed in MPA cycles. However, this finding remained within ranges reported in the literature and did not translate into inferior implantation or live birth outcomes (11, 17, 18). Mosaicism rates are known to be strong influenced by technical and laboratory-related factors, including day of biopsy (day 5 vs. day 6), number of cells analyzed, and bioinformatic thresholds applied during next-generation sequencing (17, 18). Moreover, the greater number of blastocysts biopsied per patient in the MPA group may increase the absolute detection of mosaic embryos without reflecting a true biological disadvantage.

Overall, our findings reinforce that MPA-based PPOS provides reproductive outcomes equivalent to GnRH-antagonist protocols, both in ovarian response and embryo developmental competence. Randomized trials in oocyte donation and large cohort studies have demonstrated non-inferior mature oocyte yield, fertilization, and live birth rates when comparing MPA-based protocols with GnRH antagonists (7–10). In addition, neonatal outcome studies have not identified increased risks of prematurity, low birth weight, or congenital anomalies following PPOS cycles (15, 16).

Strengths of this study include its large sample size, evaluation across two clinically relevant indications, uniform laboratory conditions, and age-stratified analyses that enhance clinical interpretability. Limitations include the retrospective design, potential selection bias, lack of neonatal follow-up within this cohort, and absence of subgroup analyses by ovarian response phenotype. As a single-center study, external validation is warranted. Future prospective randomized trials should further explore potential differences in gonadotropin requirements and mosaicism rates across ovarian reserve categories.

## 5 Conclusion

In conclusion, medroxyprogesterone acetate–based progestin-primed ovarian stimulation represents a feasible and clinically comparable alternative to gonadotropin-releasing hormone antagonist protocols for the prevention of premature luteinizing hormone surges in assisted reproductive technology. Under a uniform freeze-all strategy, MPA-PPOS provides equivalent ovarian, embryologic and clinical outcomes in both fertility preservation and ICSI-PGT-A cycles, while offering the advantages of oral administration and reduced treatment burden. These findings support the use of MPA-based oral suppression as a valid option within contemporary freeze-all ART programs.

## Data Availability

The raw data supporting the conclusions of this article will be made available by the authors, without undue reservation.

## References

[1] Mourad S Brown J Farquhar C. Interventions for the prevention of ovarian hyperstimulation syndrome in assisted reproductive technology cycles: an overview of cochrane reviews. Cochrane Database Syst Rev. (2017) 1:CD012103. 10.1002/14651858.CD012103.pub2 28111738 PMC6469542

[2] Polyzos NP Sunkara SK. Subcutaneous GnRH antagonists in IVF: convenience at a cost? Hum Reprod. (2015) 30(9):2033–5. 10.1093/humrep/dev193

[3] Kuang Y Chen Q Fu Y Wang Y Hong Q Lyu Q Progestin-primed ovarian stimulation prevents premature LH surge in IVF cycles: a randomized controlled trial. Fertil Steril. (2015) 103(1):62–69.e3. 10.1016/j.fertnstert.2015.03.022

[4] Kuang Y Chen Q Hong Q Lyu Q Ai A Fu Y Double stimulations during the follicular and luteal phases of poor responders in IVF/ICSI programmes (Shanghai protocol). Reprod Biomed Online. (2014) 29(6):684–91. 10.1016/j.rbmo.2014.08.009 25444501

[5] Roque M Haahr T Geber S Esteves SC Humaidan P. Fresh versus elective frozen embryo transfer in IVF/ICSI cycles: a systematic review and meta-analysis. Hum Reprod Update. (2019) 25(1):2–14. 10.1093/humupd/dmy033 30388233

[6] Bosch E Labarta E Crespo J Simón C Remohí J. Circulating progesterone levels and ongoing pregnancy rates in controlled ovarian stimulation cycles. Fertil Steril. (2010) 94(2):620–7. 10.1016/j.fertnstert.2009.03.004 20539042

[7] Beguería R García D Vassena R Rodríguez A. Medroxyprogesterone acetate versus ganirelix in oocyte donation: a randomized controlled trial. Hum Reprod. (2019) 34(5):872–80. 10.1093/humanrep/dez034 30927417

[8] Yu S Long H Ya H Chang N Liu Y Gao H New application of dydrogesterone as part of a progestin-primed ovarian stimulation protocol for IVF. Hum Reprod. (2018) 33(2):229–37. 10.1093/humanrep/dex367 29300975

[9] Özgür K Bulut H Berkkanoglu M Coetzee K Isikli A. Progestin-primed ovarian stimulation in assisted reproductive technology cycles: a large matched cohort study. J Assist Reprod Genet. (2020) 37(9):2305–15. 10.1007/s10815-020-01877-032623662

[10] Wang M Zheng L Ma S Xu Y Zhang J Fu L. Comparison of progestin-primed ovarian stimulation and GnRH antagonist protocols in assisted reproductive technology: a systematic review and meta-analysis. J Assist Reprod Genet. (2025) 42:3217–29. 10.1007/s10815-025-03612-4 40775480 PMC12602754

[11] Mei Y Wang Y Kuang L Lin Y Wang F. Impact of the PPOS protocol on euploidy embryo rates and reproductive outcomes in preimplantation genetic testing cycles: a systematic review. Front Endocrinol (Lausanne). (2025) 16:1595232. 10.3389/fendo.2025.1595232 40979724 PMC12446005

[12] Liu M Liu S Li L Wang P Li H Li Y. LH Levels may be used as an indicator for the time of antagonist administration in GnRH antagonist protocols—a proof-of-concept study. Front Endocrinol. (2019) 10:67. 10.3389/fendo.2019.00067 PMC6379248 30809195

[13] Ata B Kalafat E. Progestin-primed ovarian stimulation: for whom, when and how? Reprod Biomed Online. (2024) 48(2):103639. 10.1016/j.rbmo.2023.103639 38159467

[14] World Medical Association. Declaration of Helsinki: ethical principles for medical research involving human subjects. JAMA. (2013) 310(20):2191–4. 10.1001/jama.2013.281053 24141714

[15] Du M Zhang J Ren B Guan Y Comparison of the neonatal outcomes of progestin primed ovarian stimulation and flexible GnRH antagonist protocols: a propensity score-matched cohort study. Front Endocrinol (Lausanne). (2023) 14:1156620. 10.3389/fendo.2023.1156620 37396165 PMC10313097

[16] Li D Hu Z Chen Q Chai W Cai R Kuang Y Neonatal outcomes and congenital malformations in live-born infants after progestin-primed ovarian stimulation versus GnRH antagonist cycles: a multicenter cohort study. Front Endocrinol (Lausanne). (2022) 13:1062734. 10.3389/fendo.2022.1062734 PMC9681814 36440198

[17] Treff NR Marin D. The “mosaic” embryo: misconceptions and misinterpretations in preimplantation genetic testing for aneuploidy. Fertil Steril. (2021) 116(5):1205–11. 10.1016/j.fertnstert.2021.06.027 34304887

[18] Victor AR Tyndall JC Brake AJ Lepkowsky LT Murphy AE Griffin DK One hundred mosaic embryos transferred prospectively in a single clinic. Fertil Steril. (2019) 111(2):280–93. 10.1016/j.fertnstert.2018.10.019 30691630

